# Epobis is a Nonerythropoietic and Neuroprotective Agonist of the Erythropoietin Receptor with Anti-Inflammatory and Memory Enhancing Effects

**DOI:** 10.1155/2016/1346390

**Published:** 2016-11-21

**Authors:** Oksana Dmytriyeva, Stanislava Pankratova, Irina Korshunova, Peter S. Walmod

**Affiliations:** ^1^Laboratory of Neural Plasticity, Department of Neuroscience and Pharmacology, Faculty of Health and Medical Sciences, University of Copenhagen, Copenhagen, Denmark; ^2^Research Laboratory for Stereology and Neuroscience, Bispebjerg-Frederiksberg Hospital, Copenhagen University Hospital, Copenhagen, Denmark; ^3^Juliane Marie Centret, Rigshospitalet (Copenhagen University Hospital), Copenhagen, Denmark

## Abstract

The cytokine erythropoietin (EPO) stimulates proliferation and differentiation of erythroid progenitor cells. Moreover, EPO has neuroprotective, anti-inflammatory, and antioxidative effects, but the use of EPO as a neuroprotective agent is hampered by its erythropoietic activity. We have recently designed the synthetic, dendrimeric peptide, Epobis, derived from the sequence of human EPO. This peptide binds the EPO receptor and promotes neuritogenesis and neuronal cell survival. Here we demonstrate that Epobis* in vitro *promotes neuritogenesis in primary motoneurons and has anti-inflammatory effects as demonstrated by its ability to decrease TNF release from activated AMJ2-C8 macrophages and rat primary microglia. When administered systemically Epobis is detectable in both plasma and cerebrospinal fluid, demonstrating that the peptide crosses the blood-brain barrier. Importantly, Epobis is not erythropoietic, but systemic administration of Epobis in rats delays the clinical signs of experimental autoimmune encephalomyelitis, an animal model of multiple sclerosis, and the peptide has long-term, but not short-term, effects on working memory, detected as an improved social memory 3 days after administration. These data reveal Epobis to be a nonerythropoietic and neuroprotective EPO receptor agonist with anti-inflammatory and memory enhancing properties.

## 1. Background

Erythropoietin (EPO) is a secreted glycoprotein mainly produced in the kidney and liver. It serves as a cytokine strongly upregulated by hypoxia and stimulates erythropoiesis by binding to EPO receptors localized on erythroid progenitor cells. The erythropoietic property of EPO is utilized clinically for the treatment of, for example, anemia caused by renal failure or chemotherapeutic cancer therapy (reviewed by [1]). However, EPO and the EPO receptor also have additional functions. They are widely expressed in the brain, where EPO has anti-inflammatory, antiapoptotic, and neuroprotective effects, protects against oxidative stress and excitotoxicity, and stimulates angiogenesis and neurogenesis (reviewed by [2, 3]). Consequently, EPO has been evaluated for the treatment of several diseases in the nervous system including neurodegenerative diseases like Parkinson's disease [4] and multiple sclerosis [5, 6] and various types of ischemia including carbon monoxide poisoning [7, 8], subarachnoid hemorrhage [9], and Friedreich's ataxia [10]. Moreover, EPO has beneficial effects on memory and mood of animals and humans with depression-like symptoms [11]. Animal studies using EPO or EPO derivatives have demonstrated an improved memory and a reduced endothelial and neuronal degeneration in models of Alzheimer's disease [12–14], a protection against experimental cerebral malaria and pneumococcal meningitis [15, 16], downregulation of proinflammatory cytokines and upregulation of anti-inflammatory cytokines in a model of amyotrophic lateral sclerosis [17], a reduction of progressive retinal degeneration [18], and neuroprotective effects in relation to* status epilepticus* [19] and cervical subacute spinal cord compression [20]. Importantly, most of these beneficial effects of EPO are believed to be unrelated to the erythropoietic effects of EPO but not necessarily unrelated to signaling through the EPO receptor [1–3].

There are two problems associated with the systemic administration of EPO for the treatment of diseases in the nervous system: one problem is the poor transport of the protein across the blood-brain barrier (BBB) [7]; another problem is that the erythropoietic activity of EPO can cause polycythemia and thereby increase the risk of thrombosis or other adverse effects [9]. To circumvent these problems attempts have been made to design EPO derivatives that more readily cross the BBB and/or have reduced erythropoietic activities. One EPO derivative able to cross the BBB has been formed by generating a chimeric protein consisting of EPO fused to an antibody against the transferrin receptor [21]. Nonerythropoietic EPO derivatives include the mutants EpoR76E and EpoS71E (see [22]), carbamylated EPO (CEPO), EPO with low or no sialylation (neuro-EPO and asialoEPO, resp.) [23], and the peptides pHBSP/ARA290 [24, 25] and Epotris [26].

Structurally, EPO consists of 4 *α*-helices (A–D) linked by loops. A single EPO molecule interacts with predimerized EPO receptors via 2 separate binding sites: binding site 1, which is located in helices A, B, and D and a part of the AB loop, and binding site 2, located in helices A and C [27].

We have previously designed two synthetic, dendrimeric peptides with sequences corresponding to the first and the second EPO receptor binding site of human EPO, respectively. The peptide derived from binding site 2 was named Epotris and has previously been reported to promote neuritogenesis and neuronal survival* in vitro* and* in vivo* in a nonerythropoietic manner [26] and to attenuate* status epilepticus* [28]. The peptide derived from binding site 1 (residues 36–53 in the structural model of the EPO:EPO receptor-complex; PDB: 1EER [27]) was named Epobis, and surface plasmon resonance analysis has revealed that this peptide sequence specifically binds the EPO receptor with apparent *K*_D_ around 60 nM. Moreover, the peptide induces neuritogenesis in primary cultures of cerebellar granule neurons and hippocampal neurons in a dose-dependent and EPO receptor-dependent manner. Epobis also protects against neuronal cell death* in vitro*, and, like EPO, it promotes activation of the transcription factor STAT5 [29].

Here, we present data suggesting that Epobis is a nonerythropoietic and neuroprotective EPO receptor agonist with anti-inflammatory and memory enhancing properties.

## 2. Methods

### 2.1. Peptide

The Epobis peptide sequence, NENITVPDTKVNFYAWKR, corresponding to amino acids 63–80 of human EPO (Uniprot P01588) was synthesized as a tetramer (Schafer-N, Copenhagen, Denmark) as described previously [29].

### 2.2. Neurite Outgrowth from Primary Motoneurons

Primary rat motoneurons were isolated as described previously [30]. Briefly, the ventral horns of the lumbar spinal cord were dissected from Wistar rat embryos (E15). The dissociated motoneurons were plated at a density of 7,000 cells per well on laminin-coated (5 *μ*g/mL; Sigma-Aldrich) 8-well LabTek Permanox slides (NUNC, Denmark). Neurons were stimulated with serially diluted Epobis peptide for 24 h, fixed with 4% formalin, stained with polyclonal rabbit anti-rat growth-associated protein-43 antibodies (1 : 1000; Millipore), and analyzed by computer-assisted fluorescence microscopy as described [29].

### 2.3. Macrophage Activation Assay

The macrophage activation assay is used to estimate tumor necrosis factor (TNF) secretion from the macrophage cell line AMJ2-C8 (ATCC; Boras, Sweden). Fibroblastoid mouse L929 cells (L-cells; ECCC, Salisbury, UK) are sensitive to TNF upon exposure to actinomycin D, and L-cell survival can therefore be utilized as an indirect estimate of TNF concentrations [31]. The assay was performed essentially as described previously [32, 33]. Briefly, AMJ2-C8 cells were plated in 6-well MultiDish plates (Nunc, Denmark) and treated with 100 *μ*M hydrocortisone (Sigma-Aldrich, Brøndby, Denmark), 0.3 ng/mL (8.4 nM) rhEPO (Calbiochem, Merck Millipore, Denmark), or Epobis (0.9–8.1 *μ*M) for 24 h. They were then exposed to 10 ng/mL interferon-*γ* (IFN-*γ*) (R&D Systems, UK) for 24 h to induce the production of TNF [34].

L-cells were seeded in 96-well Multiwell plates (2 × 10^5^ cells/mL, 100 *μ*L/well; Nunc) and grown for 24 h. Then conditioned medium from stimulated macrophages was collected and added to the L-cells together with actinomycin D (Sigma, Sigma-Aldrich). L-cell viability was assayed 24 h later by the MTS assay (Promega, Madison, WI, USA) according to the manufacture's protocol. Cell viability was calculated as follows:

(1) $$\begin{array}{c} cell\;\;viability\;\left(\;\%\;\right)=\left(\;\frac{{OD}_{experiment}}{{OD}_{control}}\;\right)\times 100. \end{array}$$

Estimates of TNF concentrations in the supernatants from AMJ2-C8 cells were based on standard dose-response curves for L-cell viability made using recombinant mouse TNF (R&D System, MN, USA).

### 2.4. Preparation of Rat Primary Microglia Cell

Mixed glial cultures were prepared from 1-day-old to 2-day-old Wistar rat pups as described previously [35]. Briefly, cortices were dissected, roughly chopped, and incubated in prewarmed 0.5% trypsin for 10 min. Then Dulbecco's modified Eagle's medium (DMEM) plus GlutaMAX (culture media; Gibco) containing 10% fetal calf serum, penicillin (100 U/mL), and streptomycin (100 U/mL) was added and tissue was triturated. The suspension was centrifuged at 500 ×g for 10 min, and the resulting pellet was resuspended in culture media and plated in a poly-L-lysine-coated T180 flask. The mixed glial culture was grown for 2-3 weeks at 37°C in a humidified 5% CO_2_ : 95% air environment. The medium was changed first at day 2 after culture preparation and then every 5-6 days. After 2-3 weeks, loosely attached microglia cells were shaken off (250 RPM, 4 h at 37°C); medium was collected and centrifuged for 10 min (500 ×g at 4°C). The resuspended microglia cells were plated onto 24-well plates (2.5 × 10^5^ cell/mL/well) and incubated for 1-2 days. The purities of microglial cultures were confirmed in a separate experiment by staining cells with a rabbit anti-Iba-1 antibody (1 : 2000; Wako) and counterstaining with DAPI-containing mounting media (Molecular Probes). Images were recorded using a Zeiss Axiovert 100 microscope mounted with an AxioCam MRm camera using the accompanying ZEN 2012 software.

To induce TNF secretion, microglia cells were treated with lipopolysaccharide (LPS; 100 ng/mL; Sigma) following 1 h pretreatment in the absence or presence of Epobis. Conditioned media were collected 24 h later and released TNF was determined using a rat TNF Ready-SET-Go!® ELISA kit according to the manufacturer's instructions (eBioscience, San Diego, USA).

### 2.5. *In Vivo* Experiments

All* in vivo *experiments were performed according to European Union legislations and with a license from the Danish Animal Experiments Inspectorate (2008/561-1539).

#### 2.5.1. Detection of Epobis in Plasma and Cerebrospinal Fluid

Detection of biotinylated Epobis in plasma was performed essentially as described previously [26]. Briefly, 10 mg/kg Epobis was injected subcutaneously (s.c.) in anaesthetized 200 g Wistar rats, and blood samples were collected from the orbital plexus at selected time points. Cerebrospinal fluid (CSF) was sampled from the cisterna magna as described previously [36].

Subsequently the concentrations of peptide in serum plasma and CSF were estimated using a competitive enzyme-linked immunosorbent assay, where all samples were run in duplicate [26, 36].

#### 2.5.2. Hematopoiesis Assay

Estimates of hematopoietic activity of Epobis in female C57BL/6J mice were performed essentially as described previously [26]. Briefly, Epobis (10 mg/kg), EPO (10 mg/kg; Calbiochem), or vehicle (PBS) was injected subcutaneously twice per week for 5 weeks. Blood samples were collected from the orbital plexus once per week, and hemoglobin levels and hematocrit values were determined as described [26].

#### 2.5.3. Experimental Autoimmune Encephalomyelitis (EAE)

Experimental autoimmune encephalomyelitis (EAE) was induced as described previously [33]. Briefly, female Lewis rats (200 g) received subcutaneous injections at both sides of the base of the tail with 0.2 mL of an emulsion containing 1 g/L Guinea pig myelin basic protein and 1 g/L* M. tuberculosis* in Freund's complete adjuvant (all from Sigma-Aldrich). The injections were performed under full inhalation anesthesia. EAE was induced in 30 animals; 6 healthy, age-matched, unimmunized rats were used as controls. Between day 0 and day 21, the weight and clinical signs of EAE were recorded daily for all animals. Clinical signs were scored as follows: 0, no abnormality; 0.5, weak tail; 1, limp tail; 2, mild palsy of one or both hind legs; 3, severe palsy of one or both hind legs; 4, complete paralysis of one or both hind legs; 5, paralysis of one or both hind legs and beginning paralysis of front legs; 6, moribund. Animals with a clinical score ≥4 were sacrificed immediately.

Starting at day 10 after induction of EAE, animals were treated once/day for 5 consecutive days with Epobis (10 mg/kg, 1 mL/kg, s.c.) or PBS (1.0 mL/kg, s.c.). The control group is identical to the control group of previously published data [33] performed simultaneously with the Epobis study.

#### 2.5.4. Social Recognition Test

The social recognition assay evaluates short-term/working memory of adult rats, a type of memory known to be impaired in old (>18 month) rats [37] and in rats experiencing early stage AD [38]. Social recognition in rats was evaluated essentially as described previously [39]. First, a habituation session was carried out; 24 h later, Epobis (10 mg/kg, 1 mL/kg, s.c.) or PBS (1.0 mL/kg, s.c.) was administered, and 1 h later the test animals were introduced to a new, juvenile male rat for 4 min (initial trial). After another 2.5 h the same juvenile rat was reintroduced, or as a control, an unfamiliar juvenile rat was introduced (test trial). To test long-term effects of the peptide, another social recognition test was performed with the initial trial taking place 73 h after administration of test compounds. Data are expressed as a recognition ratio: RR = *T*2/(*T*2 + *T*1), where *T*1 and *T*2 are the times spent on investigating the juvenile animal during the initial and the test trial, respectively. An RR not significantly different from 0.5 suggests that the test animal has retained no memory of the introduced animal between the two meetings, whereas an RR significantly lower than 0.5 indicates that the test animal remembers the juvenile animal when introduced to the animal for the second time.

## 3. Results

### 3.1. Epobis Stimulates Neurite Outgrowth from Motor Neurons* In Vitro*

**Figure 1:**
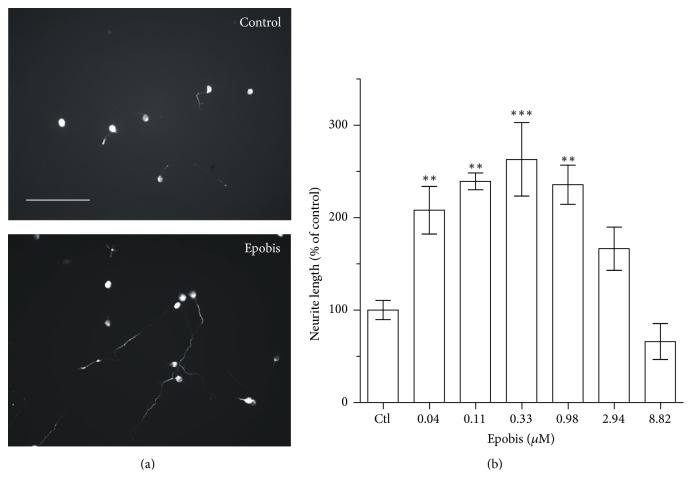
Effects of Epobis on neurite outgrowth from motor neurons. (a) Representative fluorescence micrographs of rat motoneurons grown for 24 h in the absence (top) or presence (bottom) of 0.33 *μ*M Epobis. Size bar: 10 *μ*m. (b) Neurite outgrowth in response to Epobis. The graph shows mean and SEM from 3 independent experiments. Statistics was performed on nonnormalized data using a one-way ANOVA for repeated measures (*F*_8,12_ = 20.3, *p* < 0.0001) followed by Tukey's Multiple Comparison Test. ^*∗∗*^ *p* < 0.01, and ^*∗∗∗*^ *p* < 0.001.

To evaluate whether Epobis also has neuritogenic effects in the peripheral nervous system, primary cultures of rat motor neurons were exposed to the peptide in a range of concentrations. Epobis induced a bell-shaped, concentration-dependent stimulation of neurite outgrowth (*p* < 0.0001) with maximal stimulation at 0.33 *μ*M (Figure 1).

### 3.2. Epobis Reduces Release of TNF

Activation of microglia and other macrophages leads to an increased secretion of proinflammatory cytokines including TNF. Therefore, the effects of EPO and Epobis on TNF secretion from AMJ2-C8 macrophages were detected indirectly from the survival of L-cell in response to conditioned medium from the activated macrophages.

**Figure 2:**
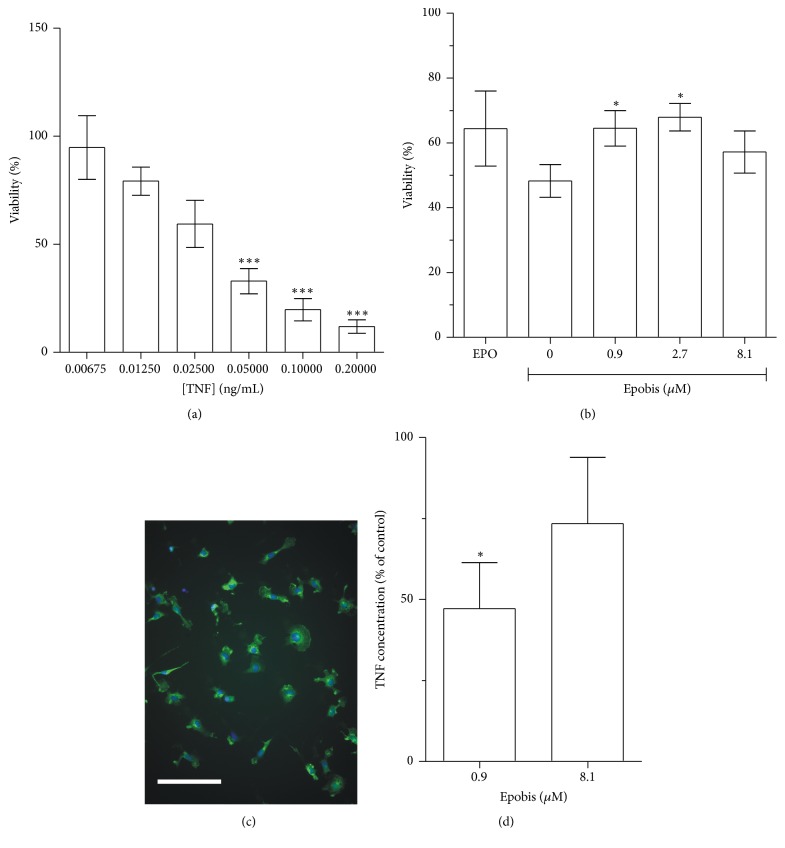
Effects of Epobis on TNF secretion. (a) Standard curve for the viability of cytokine-sensitive L-cell relative to the concentration of recombinant TNF in the medium. The curve shows mean and SEM of 4 separate experiments. L-cell viability was evaluated using a one-way ANOVA for repeated measures (*F*_5,18_ = 14.96, *p* < 0.0001) followed by Tukey's Multiple Comparison Test (^*∗∗∗*^ *p* < 0.001, when compared to 0.00675 ng/mL TNF). (b) L-cell viability in response to exposure to conditioned medium from AMJ2-C8 macrophages. A value of 100% corresponds to the viability of L-cells exposed to conditioned medium from unstimulated AMJ2-C8 cells. EPO was tested at a concentration of 8.4 nM. Data from 4 separate experiments evaluated using one-way ANOVA for repeated measures (*F*_6,9_ = 5.959, *p* < 0.0160) followed by Tukey's Multiple Comparison Test. ^*∗*^ *p* < 0.05 when compared to 0 *μ*M Epobis. (c) Representative fluorescence micrograph of the rat microglia cultures utilized for estimates of TNF secretion. The cells are double-stained with DAPI (for visualization of the total number of cells) and an antibody against Iba-1/AIF-1 (for the visualization of microglia). Size bar: 10 *μ*m. (d) Estimate of TNF secretion from primary cultures of rat microglia. The bars show mean and SEM from 3 separate experiments evaluated using Friedman's test (*p* < 0.0278) followed by Dunn's Multiple Comparison Test. ^*∗*^ *p* < 0.05, when compared to LPS-stimulated control untreated with Epobis.

A standard curve of L-cell viability relative to increasing concentrations of recombinant TNF was plotted for each individual experiment (Figure 2(a)). Conditioned medium from macrophage cultures treated with either EPO or Epobis improved the survival of the L-cells, indicative of a decreased TNF secretion from macrophages exposed to EPO and Epobis (Figure 2(b)). L-cells exposed to conditioned medium from macrophages untreated with EPO or Epobis exhibited viability of ~48% relative to control. Treatment with 8.4 nM EPO resulted in a significantly higher viability of ~64%, whereas the highest viability (~68%) in response to treatment with Epobis was obtained at a concentration of 2.7 *μ*M (*p* < 0.05; Figure 2(b)).

To verify the observed effect of Epobis on cytokine secretion in a more direct manner, TNF release was measured in primary cultures of rat microglia. First, the purity of the cultures was estimated from fluorescence micrographs of triple-stained cells, which demonstrated that 80% of the cells in the cultures were microglia (Figure 2(c)). Microglia stimulated with LPS for 24 h became activated and produced soluble TNF. It was found that treatment with 0.9 *μ*M, but not 8.1 *μ*M, Epobis caused a significant decrease in LPS-induced TNF secretion from microglia cells (*p* < 0.05; Figure 2(d)).

### 3.3. Epobis Crosses the Blood-Brain Barrier

**Figure 3:**
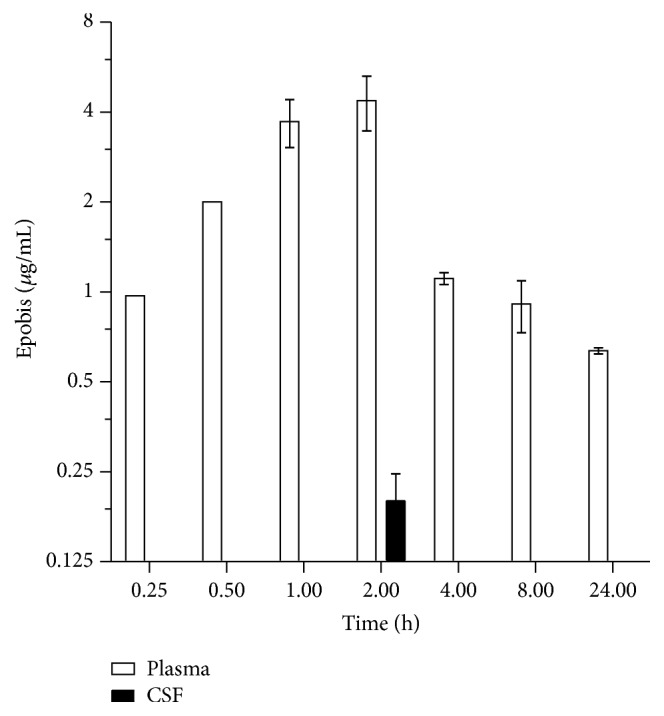
Dynamics of Epobis concentrations in plasma. Rats received a single c.s. injection of Epobis (10 mg/kg). Subsequently blood samples were collected at 15 min, 30 min, 1 h, 2 h, 4 h, 8 h, and 24 h; CSF samples were collected only 1 h after administration. The concentrations of Epobis in plasma and CSF (*n* = 6) were estimated by ELISA.

To test the distribution and dynamics of Epobis in response to systemic administration, the concentrations of the peptide in blood and CSF were measured in rats following a single s.c. injection of biotinylated Epobis. The peptide was detectable already at the time of the first blood sample (15 min after administration) and remained detectable in the blood for at least 24 h. However, the plasma concentration of Epobis peaked around 2 h after administration and decreased rapidly between 2 and 4 h after administration (Figure 3). In order to determine whether Epobis is capable of crossing the BBB, cerebrospinal fluid (CSF) samples were collected 2 h after a single s.c. peptide administration. The obtained results revealed that the peptide was present in the CSF at a concentration of 0.2 *μ*g/mL, which was approximately 20 times lower than the plasma concentration at the same time point (Figure 3).

### 3.4. Epobis Has No Hematopoietic Activity

**Figure 4:**
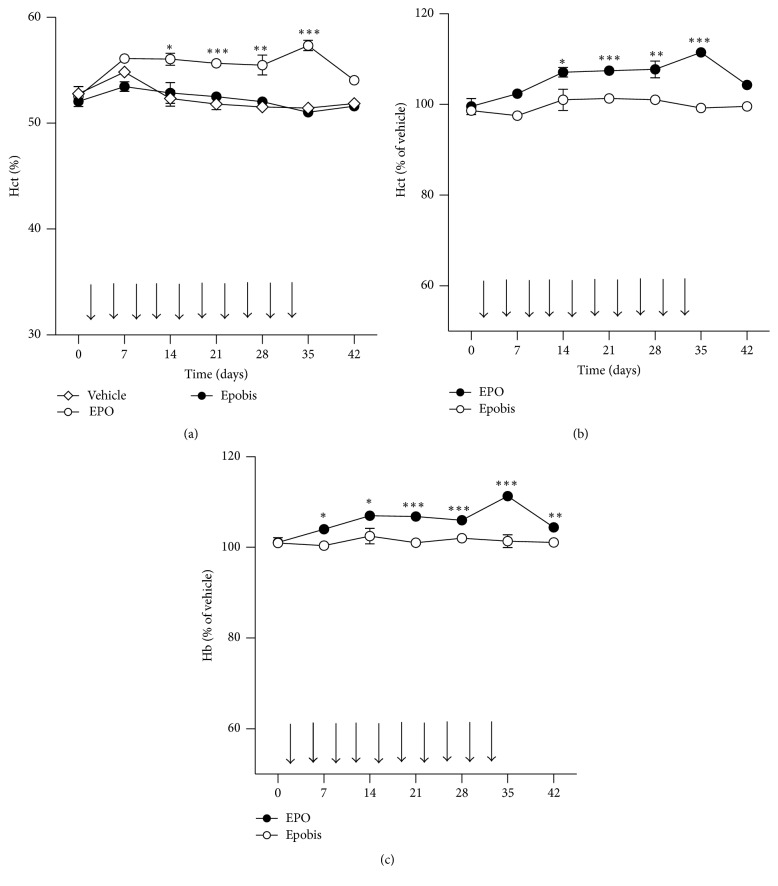
Effects of Epobis on hematopoiesis* in vivo*. Mice were injected with Epobis (*n* = 8), EPO (*n* = 10), or PBS (*n* = 6) twice per week for 5 weeks (black arrowheads). Hematocrit (a, b) and hemoglobin levels (c) were estimated from blood samples taken once per week for 6 weeks; first sample day, 0; last sample day, 42. Data are presented as actual values (a) and normalized to vehicle (PBS) from the same day (100%, b and c). Statistics was performed on nonnormalized data using a one-way ANOVA for repeated measures followed by Tukey's Multiple Comparison Test. ^*∗*^ *p* < 0.05; ^*∗∗*^ *p* < 0.01; ^*∗∗∗*^ *p* < 0.001, Epobis versus vehicle.

To test their hematopoietic properties* in vivo*, EPO or Epobis was administered s.c. to mice twice per week over a period of 5 weeks, and blood samples were collected once per week. Administration of EPO caused statistically significant increases in both hematocrit (Figures 4(a) and 4(b)) and hemoglobin levels (Figure 4(c)). More than 1 week after the administration of EPO had been terminated the hemoglobin levels, but not the hematocrit levels, of EPO-treated mice were still significantly different from the level prior to EPO administration. In contrast, Epobis did not cause any statistically significant increases in hematocrit or hemoglobin levels at any time point investigated (Figures 4(a)–4(c), filled symbols).

### 3.5. Epobis Delays the Clinical Signs of Experimental Autoimmune Encephalomyelitis

**Figure 5:**
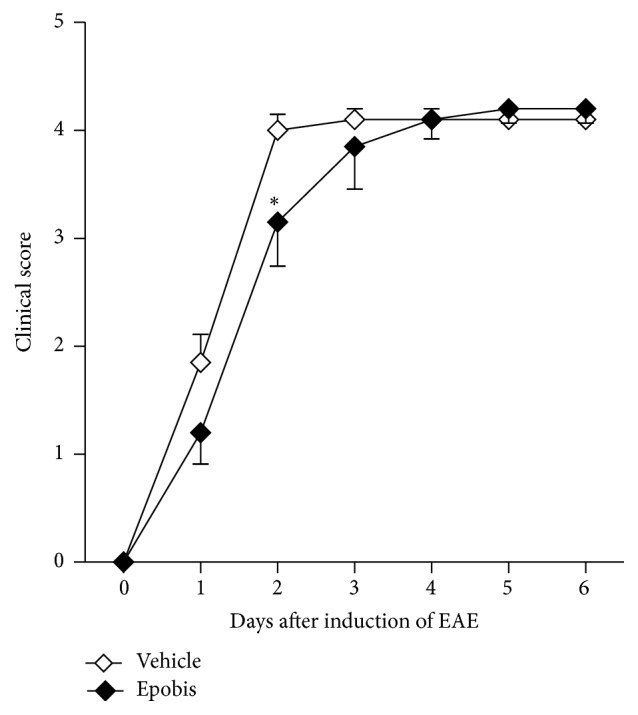
Effects of Epobis on the clinical signs of EAE. Following induction of EAE the clinical signs were evaluated daily for all animals. Only animals reaching a clinical score ≥1 before day 14 were included in the study. EAE was induced in 30 animals of which 20 developed clinical signs of EAE (10 receiving PBS; 10 receiving Epobis). In the figure, the data have been aligned according to the onset of clinical signs that were scored as follows: 0, no abnormality; 0.5, weak tail; 1, limp tail; 2, mild palsy of one or both hind legs; 3, severe palsy of one or both hind legs; 4, complete paralysis of one or both hind legs; 5, paralysis of one or both hind legs and beginning paralysis of front legs; 6, moribund. Animals with a clinical score ≥4 were sacrificed immediately. The clinical scores of animals sacrificed due to a clinical score ≥4 have been maintained at all subsequent time points. Data are shown as mean and SEM. The data were evaluated using a 2-way ANOVA for repeated measures followed by a Bonferroni posttest. ^*∗*^ *p* < 0.05 relative to the corresponding control animals.

To test the anti-inflammatory properties of Epobis* in vivo*, the peptide was administered to rats undergoing EAE, an autoinflammatory animal model of multiple sclerosis [40]. Animals undergoing EAE demonstrated significant weight loss, but Epobis had no significant effects on the weight changes or the survival of EAE animals when compared to vehicle (see Additional File 1 in Supplementary Material available online at http://dx.doi.org/10.1155/2016/1346390). Immunohistochemical analysis demonstrated an increased number of activated microglia in the brain stem of EAE animals when compared to control, whereas no difference could be observed between EAE animals receiving Epobis or vehicle, respectively (see Additional File 2). However, Epobis appeared to delay the increase in the degree of the clinical signs of EAE. In Figure 5, the clinical signs recorded for the individual animals have been aligned according to their first appearance. The clinical signs for Epobis-treated animals increased less rapidly than for vehicle-treated animals, and at day 2 after onset of clinical signs, the clinical signs for Epobis-treated animals were significantly lower than those for vehicle-treated animals (*p* < 0.05). However, at later time points, the clinical signs in Epobis-treated animals were not significantly different from those of vehicle-treated animals.

### 3.6. Epobis Improves Social Memory

**Figure 6:**
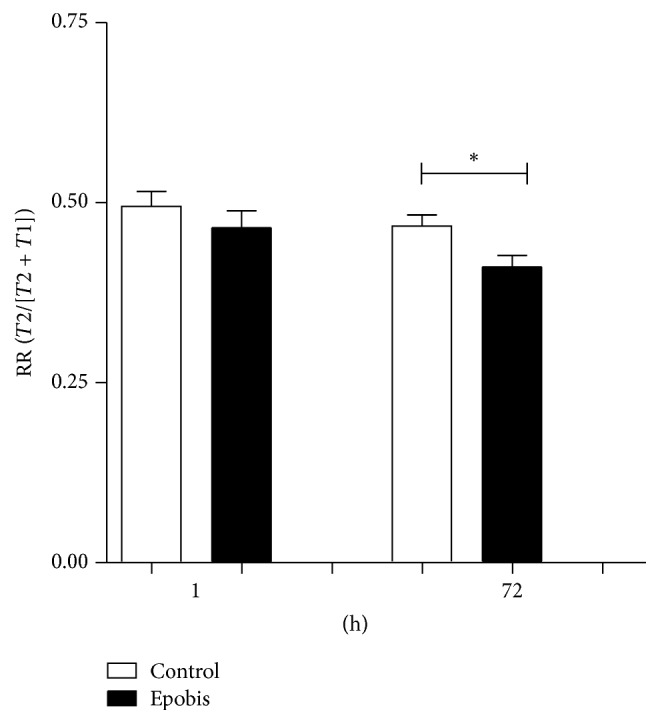
Effects of Epobis on social memory. Healthy adult rats received a single administration of PBS (open columns, *n* = 12) or Epobis (solid columns, *n* = 12), and social recognition tests were initiated 1 h and 73 h later. Data are expressed as a recognition ratio: RR = *T*2/(*T*2 + *T*1), where *T*1 and *T*2 are the times spent on investigating the juvenile animal during the initial and the test trial, respectively. Data were evaluated using* t-*tests. ^*∗*^ *p* < 0.05 relative to the corresponding control animals.

The effects of Epobis on short-term/working memory were tested using the social recognition test on healthy adult rats. When performing the social recognition test 1 h after administration of test compounds, there was no significant difference between the RR of animals treated with Epobis and vehicle, respectively (*t*-test, *p* > 0.05), and in both cases the RR was not significantly different from 0.5 (*t*-test, *p* > 0.05 for both control and Epobis). However, when performing the social recognition test 73 h after administration of test compounds, the RR of Epobis-treated animals was significantly lower than that of vehicle-treated animals (*t-*test, *p* < 0.018) and significantly different from 0.5 (see Materials and Methods;* t*-test, *p* > 0.05 and *p* < 0.0001 for control and Epobis, resp.; Figure 6).

## 4. Discussion

Here it is shown that Epobis can induce bell-shaped, concentration-dependent stimulation of neurite outgrowth in cultures of primary motoneurons. Moreover, Epobis has anti-inflammatory effects* in vitro *as demonstrated by an ability to decrease TNF secretion from macrophages. Following systemic administration the peptide is able to cross the BBB, but in contrast to EPO no hematopoietic activity* in vivo *could be demonstrated for the peptide. An analysis of the anti-inflammatory effects of Epobis* in vivo* as evaluated using the EAE model suggests that the peptide (10 mg/kg, 1 mL/kg, s.c.) with 1 daily administration starting at day 10 after induction of EAE can delay but not prevent the clinical signs of EAE. Finally, a single administration of Epobis (10 mg/kg, 1 mL/kg, s.c.) has long-term effects leading to an improved social recognition memory 3 days after administration.

Epobis has previously been demonstrated to stimulate neurite outgrowth in cultures of cerebellar granule neurons and hippocampal neurons [29]. The data demonstrating also an effect on the neurite outgrowth from motoneurons suggest that the peptide can act on neurons of both the peripheral and central nervous system. However, a prerequisite for Epobis to target neurons is an ability to penetrate the BBB. Therefore,* in vivo *experiments with Epobis were initiated. When administered systemically only a very small fraction of recombinant EPO crosses the BBB [7]. Peptides generally penetrate the BBB more readily than glycosylated proteins, and therefore EPO-mimetic peptides could potentially enter the CNS easier than recombinant EPO. Indeed, both the peptides derived from the EPO:EPO receptor binding site 2, Epotris [26] and Epobis, tested here, penetrate the BBB. The plasma : CSF ratio for Epobis 2 h after administration was ~20 (Figure 3). This value is comparable to those from previous studies with other peptides demonstrating ratios of ~10–55 [32, 33, 36, 38].

EPO is known for its erythropoietic potential [1], a function considered counterproductive for the neuroprotective potential of the protein [3]. Therefore, in addition to potentially improved penetration of the BBB, the main reason for designing EPO mimetics is to create compounds that have the same neuroprotective properties as EPO but not its erythropoietic properties. Although Epobis binds to and signals through the EPO receptor the peptide could not be demonstrated to have any erythropoietic properties in mice when tested at the same administration schedule utilized in other* in vivo* experiments (Figure 4). These data are comparable to those obtained with Epotris, which at doses between 10 and 40 mg/kg was unable to promote significant changes in hematocrit and hemoglobin levels [26].

The neuroprotective actions of EPO are known in part to be a result of anti-inflammatory effects [3], including a reduction in the production of the proinflammatory cytokine TNF [41–43]. For example, the protein can attenuate the immune response triggered by CNS injury, partially by decreasing TNF levels [44, 45]. Therefore, the anti-inflammatory potential of Epobis was investigated* in vitro*. As expected, exposure to EPO caused a significant reduction in TNF secretion from macrophages and also Epobis reduced TNF release from macrophages, thus further demonstrating that the peptide has an anti-inflammatory potential* in vitro*.

Subsequently, the effects of Epobis were evaluated* in vivo* on the progression of EAE in rats. EAE is a classical model for multiple sclerosis, and in addition to specific anti-inflammatory effects, EPO and EPO derivatives are in this model believed to have beneficial effects due to antioxidative and other neuroprotective actions [46, 47]. Epobis had only minor effects on the progression of EAE. The peptide caused no significant changes in the survival or the weight changes of EAE animals, and the only detected significant change observed in response to the peptide treatment was a slight delay in the progression of the clinical signs (Figure 5).

The beneficial effects of Epobis on the progression of EAE may be related to the reduced secretion of proinflammatory cytokines demonstrated* in vitro*. However, the exact mechanisms of the anti-inflammatory effects of Epobis* in vivo *would require additional studies preferentially using a model demonstrating stronger effects in response to peptide administration. An explanation for the weak effect of Epobis on EAE might be found in the experimental setup. In a study of the effects of EPO on MOG-induced EAE in mice, EPO was administered 4 times between postimmunization days 1 and 7. This treatment caused a reduction in the clinical scores, a delay in the onset of the clinical signs, and a lower incidence of EAE [48]. In another study, EPO had the most pronounced effects on the clinical signs on EAE in rats, when the protein was administered prior to the onset of the clinical symptoms as compared to when the animals already had clinical signs at the onset of EPO administration [49]. Together, these observations suggest that the effects of Epobis on the progression of EAE could have been improved by administering the peptide earlier after immunization. Moreover, the administered dose of Epobis may be further optimized. It would be interesting to perform additional studies of the effects of Epobis on EAE or other neuroinflammatory models using a different administration protocol and more than one drug concentration. Moreover, if Epobis has better* in vivo* effects on, for example, EAE using a different administration protocol, it would be interesting to relate the effects to changes in the activation of microglia and other signs of inflammation.

Since EPO and EPO derivatives are known to have beneficial effects on memory formation [4, 11, 13, 14, 16, 40, 50–52], it was evaluated whether Epobis also can improve memory formation in healthy animals. Epobis was found to improve the social recognition of healthy adult rats 3 days, but not 1 h, after treatment (Figure 6), suggesting that Epobis-induced changes in gene transcription and other downstream effects lasting more than 1 h are required for the beneficial effects on memory to appear or that the effects on memory peak several days after Epobis exposure. The result can also be explained by the fact that Epobis needs time to cross BBB and reach neuronal targets, since we have showed here that this peptide peaks in plasma only 2 h after peripheral injections. The mechanism for the Epobis-improved memory of healthy animals is unclear, since most studies of the effects of EPO and EPO derivatives on memory have been performed on animals exposed to a brain injury or undergoing a disease model. However, in a previous study of healthy young mice, the beneficial effects on memory of long-term EPO treatment were found to be caused by effects on synaptic plasticity, connectivity, and activity of hippocampal neuronal networks [40] and a similar effect may explain the beneficial effects of Epobis.

## 5. Conclusions 

The synthetic, dendrimeric peptide, Epobis, derived from the sequence of human EPO has previously been demonstrated to be an EPO receptor agonist facilitating neuritogenesis and neuronal survival* in vitro* [29]. Here it is shown that it has anti-inflammatory effects* in vitro* as demonstrated by an ability to decrease TNF secretion from AMJ2-C8 macrophages and rat primary microglia with an efficacy comparable to, but with a lower potency, EPO.* In vivo* Epobis is able to cross the BBB leading to a plasma : CSF ratio of ~20, 2 h following systemic administration. Even after long-term treatment, the peptide has no hematopoietic activity* in vivo*. Acute treatment of EAE with a single daily injection of Epobis delays but does not prevent the clinical signs of EAE. Finally, Epobis has long-term but not short-term effects on the social recognition memory of rats, leading to an improved memory 3 days after administration. Thus, Epobis is a novel EPO receptor agonist potentially suitable for the treatment of neurodegenerative diseases.

## Supplementary Material

Supplementary Figure 1 presents curves of weight changes and survival for control animals (rats) and EAE-induced animals treated or untreated with Epobis. Supplementary Figure 2 presents histochemical stainings of microglia in spinal cords from ontrol animals (rats) and EAE-induced animals treated or untreated with Epobis. The data show no significant changes between EAE-induced animals treated or untreated with Epobis.

## Abbreviations

BBB: Blood-brain barrier
CEPO: Carbamylated EPO
CSF: Cerebrospinal fluid
EAE: Experimental autoimmune encephalomyelitis
EPO: Erythropoietin
IFN: Interferon
TNF: Tumor necrosis factor.
